# An Investigation of the Modulating Effects of Sensory Stimulation and Transcranial Magnetic Stimulation on Memory-Related Brain Activity

**DOI:** 10.3390/brainsci15111182

**Published:** 2025-10-31

**Authors:** Stevan Nikolin, Matthew Wang, Adriano Moffa, Haijing Huang, Mei Xu, Siddhartha Raj Pande, Donel Martin

**Affiliations:** 1 Discipline of Psychiatry and Mental Health, School of Clinical Medicine, Faculty of Medicine and Health, University of New South Wales, Sydney, NSW 2052, Australia; stevan.nikolin@unsw.edu.au (S.N.); matthew.wang@student.unsw.edu.au (M.W.);; 2 Black Dog Institute, Sydney, NSW 2031, Australia; s.pande@blackdog.org.au; 3 Department of Brain and Cognitive Sciences, Massachusetts Institute of Technology, Cambridge, MA 02139, USA

**Keywords:** episodic memory, hippocampal network, transcranial magnetic stimulation, audiovisual stimulation

## Abstract

**Background/Objectives**: As the global population ages, the prevalence of disorders associated with memory dysfunction (e.g., Alzheimer’s disease) continues to increase. There is a need for novel interventions that can enhance memory and support affected individuals. Non-invasive brain stimulation provides a promising approach to engage circuits within the hippocampal network, a group of brain regions critical for episodic memory, and thereby improve cognition. **Methods**: Twenty healthy participants completed a single-blind, within-subject crossover study over four sessions. In each session, they received one of four interventions whilst viewing pictures of real-world objects: 40 Hz synchronised audiovisual stimulation (AVS), theta burst stimulation (TBS), a combination of synchronised 5 Hz repetitive transcranial magnetic stimulation with AVS (rTMS + AVS), or sham rTMS. Electroencephalography (EEG) was recorded to measure associated brain activity changes. Following each intervention, participants completed a recognition memory task. **Results**: Mixed-effect repeated measure models (MRMMs) revealed no significant differences in recognition memory performance or theta (5 Hz) activity across conditions. However, both TBS and rTMS + AVS significantly increased gamma (40 Hz) activity compared to sham rTMS, and TBS induced a widespread increase in theta-gamma phase-amplitude coupling during picture viewing. **Conclusions**: While the neuromodulatory interventions did not enhance memory performance, the observed increase in gamma activity, particularly following rTMS-based stimulation, suggests potential engagement of neural processes associated with memory. These findings warrant further investigation into the role of gamma oscillations in memory and cognitive enhancement.

## 1. Introduction

Episodic memory involves the learning, storage, and recollection of prominent events, images, or actions [1]. It plays a crucial role in guiding interactions with our environment and informing decision-making by integrating contextual features with sensory-based memories, particularly those associated with time and place [2]. Unfortunately, episodic memory is impaired in multiple neuropsychiatric disorders (e.g., Alzheimer’s dementia, Major Depressive Disorder, Schizophrenia) and current treatments, including pharmacological and psychological treatments, have limited effects [3,4,5]. There is hence an urgent need to develop effective novel therapies which can improve episodic memory and patient functioning in people with memory impairment. Neuroimaging studies have consistently identified the hippocampal network as the relevant anatomical substrate that subserves episodic memory [6,7,8,9]. This network can be modulated through cortical entrainment, achieved by rhythmic sensory stimulation of the auditory or visual cortices using audiovisual stimuli (AVS), particularly when delivered in the gamma frequency band [10]. Similarly, non-invasive brain stimulation techniques, such as repetitive transcranial magnetic stimulation (rTMS), have demonstrated potential to entrain cortical oscillatory activity, modulate cortical excitability, and improve memory [11,12]. Both AVS and rTMS therefore show promise for non-invasively modulating hippocampal network activity and potentially enhancing episodic memory functioning. This study aimed to investigate the use of AVS, rTMS, and a novel combination of synchronised rTMS and AVS, to entrain neuronal oscillatory activity and improve episodic memory performance.

The hippocampus and interconnected brain regions, including the medial prefrontal cortex, the medial and lateral parietal cortices, and the medial temporal lobe [13,14], together form the hippocampal network [15]. An extensive body of literature has elucidated the role of the hippocampal network in the formation, encoding, and retrieval of episodic memories [16]. Theta rhythms (4–8 Hz), believed to originate from septal and hippocampal interneuron activity, as well as excitatory inputs to the hippocampus, are thought to facilitate the temporal organization of memory by supporting the encoding of sequences of events [17]. In contrast, ‘slow’ gamma activity (25–55 Hz), which is typically of lower amplitude than the theta rhythms, is thought to promote memory retrieval [17]. Importantly, recent studies have demonstrated that neural activity within the hippocampal network can be entrained and enhanced using non-invasive brain stimulation techniques [18,19,20,21,22,23,24,25,26]. 

RTMS, a non-invasive form of brain stimulation, offers a method to modulate neural activity via magnetic fields that induce electrical current in cortical tissue [27]. High frequency rTMS (HF-rTMS), i.e., protocols in which the repetitive magnetic stimulus is delivered at frequencies ≥ 5 Hz [28], has excitatory neuromodulatory effects [29,30,31]. Given that hippocampal activity is often characterized by theta-band oscillations, this frequency range has become a target for memory enhancement studies using rTMS [13,26,32,33], although interestingly, even higher frequencies, such as 20 Hz HF-rTMS, have been shown to improve episodic memory performance [19]. A more recent variation of rTMS, called theta-burst stimulation (TBS), delivers triplet bursts of 50 Hz pulses repeated at 5 Hz, mimicking endogenous synchronized theta-gamma rhythms [34]. Intermittent TBS has been shown to increase the excitability of stimulated motor neurons [34,35], whereas continuous TBS can decrease it [36,37]. These neuromodulatory effects are considered comparable to, or even greater than, those of conventional rTMS [38,39]. TBS has also been shown to effectively modulate naturally occurring brain oscillations, offering a promising avenue for entrainment [26,40]. For instance, Hermiller, Chen [26] demonstrated that TBS increased left-lateralized hippocampal network activity during visual memory encoding and retrieval, as measured by functional MRI (fMRI), suggesting a beneficial effect on episodic memory.

Rhythmic sensory stimulation is another emerging non-invasive technique with potential to entrain hippocampal network activity. Like rTMS, sensory stimulation aims to synchronize neural activity by matching endogenous oscillatory frequencies with externally delivered stimuli [41]. The most widely studied form is audiovisual stimulation (AVS), which involves rhythmic visual (e.g., flickering images) and auditory (e.g., sinusoidal tones) stimuli [10,18], which in combination activate approximately 85% of the sensory input to the brain [42]. Electroencephalography (EEG) studies in healthy populations have shown that AVS can increase frequency-specific neuronal entrainment [43,44]. Given that episodic memory relies on the integration of sensory information within the hippocampal network, AVS may be particularly well-suited for memory enhancement [1]. In contrast to rTMS, AVS has shown advantages in entraining activity at higher frequency bands relevant to cognitive functions, especially beta and gamma [18], and in stimulating deeper brain regions, including the hippocampus [45]. Indeed, in animal models of Alzheimer’s disease, gamma-frequency stimulation has been associated with improved cognitive function [46,47,48], and neuroplastic changes [47]. Moreover, recent studies have demonstrated the safety and feasibility of administering 40 Hz AVS in patients with Alzheimer’s disease, highlighting its potential for clinical application [43,45,46,49,50].

Taken together, both AVS and rTMS may effectively entrain cortical oscillatory processes and potentially improve memory. It remains unclear, however, which methodology is most effective. This study therefore aimed to investigate the potential utility of these non-invasive brain stimulation techniques, and their novel combination, for entrainment of gamma and theta cortical oscillations and improving memory. We hypothesised that all active conditions would increase gamma-band activity during memory encoding, and that the combined rTMS + AVS condition would additionally enhance theta-band activity, as compared to a sham control. Furthermore, we expected that all active conditions would improve performance on a recognition memory task compared to sham stimulation.

## 2. Materials and Methods

### 2.1. Participants

Twenty healthy participants were recruited for the study. Participants reported no history of neurological illness or brain injury, were non-smokers, had no current history of drug or alcohol abuse or dependence, had no contraindications to TMS, and were not using any medication acting on the central nervous system at the time of the study. We powered our study to detect a moderately sized effect (Cohen’s *f* = 0.3, α = 0.05, β = 0.80; ≤15% dropout; G*Power Version 3.1.9.7), in line with effect sizes reported by an investigation of TBS for memory enhancement in healthy participants [26]. The experimental procedure was approved by the University of New South Wales Human Research Ethics Committee (iRECS5059). According to the declaration of Helsinki, all participants provided written informed consent prior to the start of the experiment.

### 2.2. Experiment Design

**Figure 1:**
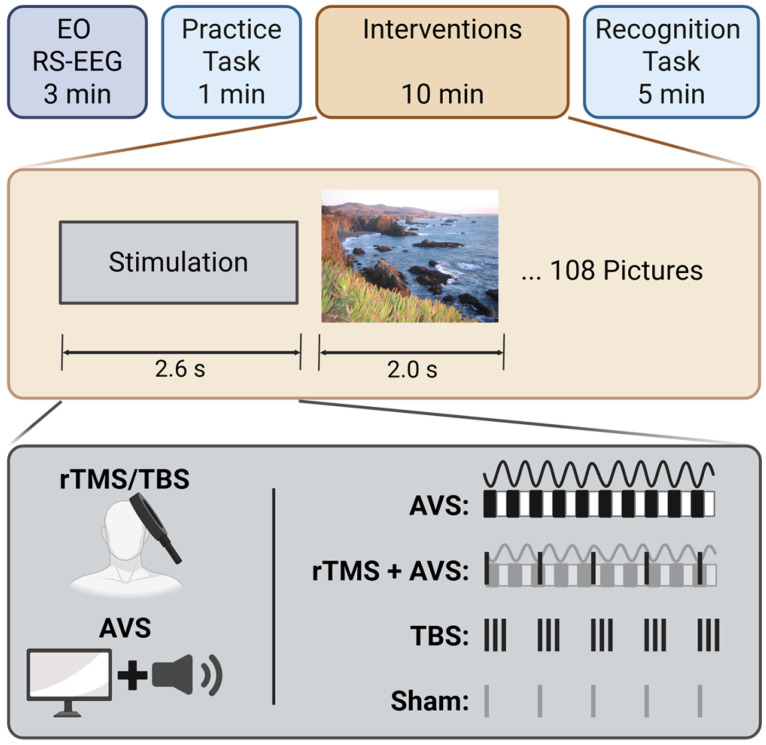
Experiment design. The experiment began with 3 min of eyes-open resting-state EEG (EO RS-EEG), followed by a brief picture practice task to familiarise participants with the procedure. During the intervention phase, participants received one of the following conditions: 40 Hz audiovisual stimulation (AVS), 5 Hz repetitive transcranial magnetic stimulation combined with AVS (rTMS + AVS), theta burst stimulation (TBS), or sham rTMS, while viewing images as part of a recognition memory task. Finally, participants completed the recognition phase, identifying 108 previously viewed target images among 108 novel lure images.

Each participant completed four sessions (AVS, rTMS + AVS, TBS, and sham rTMS) in a single-blinded crossover design, with the order of sessions randomised across participants using a 4 × 4 Latin square design. Each session began with an eyes-open resting-state EEG for 3 min, in which participants were instructed to focus on a fixation cross and to relax with minimal movement. Following this, participants completed a 1-min practice block to familiarize themselves with the recognition memory task. They were first shown five standard landscape pictures and were then asked to identify whether each image, when later presented in a randomized sequence alongside five novel art gallery lure images, was previously seen or new. After the practice block, participants viewed images from the recognition memory task over a 10-min period (see Section “Recognition Memory Task”) while receiving the neuromodulatory intervention. Finally, they completed the 5-min recognition phase of the memory task (see Figure 1). To minimise the potential for carry over effects from each of the stimulation conditions, a minimum of 1-day in-between sessions was implemented.

#### Recognition Memory Task

The recognition task was adapted from a previous study [26] and administered using Inquisit software (Version 6, Millisecond Software). Participants observed 108 pictures during the neuromodulatory intervention period, which alternated between 2.6 s of stimulation followed by a 2 s picture display. Four parallel picture libraries were used, to prevent carryover from prior sessions. During picture presentation, participants were instructed to imagine visiting the depicted location. Pictures were chosen from the SUN397 dataset [51] and included: complex outdoor natural scenes without prominent humans, animals, or man-made objects; colour images without text. Participants were told that their memory would be tested following the presentation. During the subsequent recognition phase the 108 pictures were then re-presented along with 108 novel lure pictures. Participants were required to respond if they recognised a picture by pressing the letter ‘L’, or if they believed it was a novel lure by pressing ‘A’, on a computer keyboard.

### 2.3. Interventions

#### 2.3.1. Audiovisual Stimulation (AVS)

AVS consisted of a synchronised 40 Hz audio and a flickering black and white screen at 40 Hz. The audio was administered through earphones and the flickering screen was generated using a custom MATLAB script. Monitor brightness was standardised at 400 lumens, and audio volume was adjusted to the maximum level for participant comfort and tolerability, capped at 90 dB for safety.

#### 2.3.2. Repetitive Transcranial Magnetic Stimulation (rTMS) with AVS

TMS was administered with a MagPro R30 stimulator (MagVenture A/S, Farum, Denmark) using a mcf-125 circular coil. A neuronavigation system (Visor2, ANTNeuro, Enschede, The Netherlands) and Polaris Vicra optical tracking camera (Northern Digital Inc., Waterloo, Ontario, Canada) were used to accurately position the TMS coil over a left centroparietal target. The target was operationalised as MNI coordinate (−53, −41, 27) using a default average head model. The TMS coil was placed 45 degrees to the sagittal plane with the handle pointing behind the participant and angled at 20 degrees above the tangent.

Resting motor threshold (RMT) was operationalised as the lowest stimulus intensity required to elicit at least three out of six motor-evoked potentials with a peak-to-peak amplitude of at least 50 µV across the contralateral right first dorsal interosseous muscle. EMG was measured using a 1401 laboratory interface with a 1902 amplifier (Cambridge Electronic Design, Cambridge, UK) and the Signal V4 data acquisition package (Cambridge Electronic Design, Cambridge, UK).

For the combined intervention, AVS was given in conjunction with an rTMS protocol consisting of a train of 13 pulses repeated at a rate of 5 Hz, delivered at 80% RMT.

#### 2.3.3. Theta Burst Stimulation (TBS)

The TBS protocol consisted of a train of 13 bursts of 40 Hz triplets delivered at a rate of 5 Hz, with an intensity of 80% RMT.

#### 2.3.4. Sham rTMS

To preserve blinding a TMS coil was placed on the participant’s head as described in Section 2.3.2 but did not deliver any stimulation. Instead, a secondary coil was placed vertically above the participants head to generate the sound of stimulation. Additionally, to avoid unintended frequency-specific effects using an alternative form of AVS, the control condition used a blank screen with no audio.

### 2.4. Electroencephalography Data Acquisition

Continuous EEG data sampled at 4096 Hz were acquired with an EEG amplifier (EE-225, eemagine GmbH, Berlin, Germany) and a TMS-compatible 64-electrode 10–10 system cap with sintered Ag/AgCl pins with shielded cables (waveguard™ original, ANT Neuro B.V., Hengelo, The Netherlands). Electrodes were grounded to AFz and online referenced to CPz, and impedance levels were kept at <5 kΩ throughout the experiment.

### 2.5. Electroencephalography Pre-Processing

EEG data were cleaned and analysed offline, after all experimental sessions had been completed and the dataset had been locked, using Fieldtrip [52] and custom scripts on the MATLAB platform (R2021a, The MathWorks, Natick, MA, USA).

The data from the online reference channel (CPz) were reconstructed using the average-referenced EEG data. Task-related EEG data from 100 ms prior to the neuromodulatory intervention and ending 160 ms prior to picture onset was removed due to strong artifacts induced by TMS and replaced using piecewise cubic spline interpolation. A second-order band-pass filter was applied, with cut-off frequencies set at 0.1 and 100 Hz. A Butterworth IIR digital filter was used to eliminate 50 Hz line noise. Continuous EEG data recorded during the task were segmented into epochs extending from 150 ms before to 1850 ms after picture onset. Resting-state EEG (RS-EEG) data were segmented into consecutive 2-s epochs. Following this, a manual procedure was employed to identify and reject trials and channels with significant artefacts. Rejected channels were replaced using piecewise cubic spline interpolation. Independent Components Analysis (ICA) was used to remove non-cortical physiological activity (e.g., cardiac, muscle, ocular) and non-physiological activity (e.g., environmental noise, movement). This ICA was conducted using the runica function (Infomax). A high-variance electrode artifact removal (HEAR) algorithm [53] and source-estimate-utilizing noise-discarding (SOUND) algorithm, which is used to suppress noise signals from EEG and magnetoencephalography [54], were applied to clean any remaining artefacts. Data was re-referenced using the common average reference.

### 2.6. Event-Related Synchronisation/Desynchronisation

We assessed the effects of our interventions on cortical oscillations using event-related synchronisation/desynchronisation (ERS/D). We applied a Short-Time Fourier Transform (STFT) using the MATLAB native spectrogram function with a 200 ms window, 150 ms overlap to each trial and each electrode. Spectral power was then averaged across all trials, capturing both evoked (i.e., phase-locked) and induced (i.e., non-phase-locked) activity. Due to concerns that the pre-stimulus window may contain residual effects of neuronal entrainment, baseline correction was performed using RS-EEG recording at the start of the experiment. RS-EEG was similarly epoched into 2 s segments, processed with STFT, and then averaged over epochs. Frequency-specific power during the task block was then expressed as a relative change from this RS-EEG baseline.

We focused on theta and gamma ERS/D as these reflect the stimulation parameters of our interventions. The time interval for time-frequency analysis was set at 0 to 100 ms following picture onset to capture acute neural entrainment effects. We assessed theta (4 to 8 Hz) activity in a left temporal region of interest comprising electrodes C1, C3, CP1, and CP3 to detect changes within the hippocampal network. We assessed gamma activity (38 to 42 Hz) at electrode Oz to capture entrainment within the visual cortex.

### 2.7. Phase-Amplitude Coupling

Theta-gamma phase-amplitude coupling (PAC) was quantified during a post-picture onset time interval of 500 to 1850 ms using similar methodology to prior studies [55,56]. PAC was computed using the modulation index (MI), as described by Tort, Komorowski [57], which quantifies the deviation of the amplitude distribution across phase bins from a uniform distribution using the Kullback–Leibler (KL) divergence [57].

Bandpass filtering was performed using a finite impulse response (FIR) filter. Theta-band activity was extracted from a fronto-central electrode cluster and filtered between 4–6 Hz. The instantaneous phase of the theta signal was obtained via the Hilbert transform. Gamma-band activity was filtered between 55–85 Hz, and the amplitude envelope was extracted using the absolute value of the Hilbert transform. PAC was calculated using 18 phase bins spanning the range from −π to π. For each trial, gamma amplitude values were assigned to the corresponding theta phase bin. The average amplitude within each bin was computed, resulting in a phase-amplitude distribution. This distribution was normalized to form a probability distribution, and the MI was calculated as the KL divergence between this empirical distribution and a uniform distribution, normalized by the logarithm of the number of bins.

### 2.8. Statistical Analysis

Statistical analyses were conducted using R (version 4.3.0) and MATLAB (version 2017b, MathWorks, Natick, MA, USA). Mixed effects repeated measures models (MRMMs) were implemented using the lme4 package in R. MRMMs were applied to behavioural outcomes (accuracy on picture recognition memory task) and EEG outcomes (theta and gamma ERS/D; theta-gamma PAC). Each model included ‘Condition’ (AVS, rTMS + AVS, TBS, and sham) as a fixed effect and ‘Participant’ as a random effect. Outliers were identified as values in the overall sample (i.e., ignoring Condition) exceeding 1.5 times the interquartile range. Rosner’s test was subsequently applied to evaluate whether the number of detected outliers was statistically supported by the data distribution [58]. Residuals were visually inspected to check for normality violations and ensure adequate convergence of model parameters [59]. Effect sizes were calculated using bootstrapped Cohen’s *d* comparing active conditions to the sham control.

Additionally, exploratory non-parametric cluster-based permutation tests (two-tailed) were performed on ERS/D data. Cluster-based permutation testing controls for the family-wise error rate while performing statistical comparisons across a large spatiotemporal parameter space [60,61]. Participants were randomly permuted over 5000 iterations using the Monte Carlo method. Permutation testing included data from all EEG channels within a 0 to 1000 ms time window following picture onset. Analyses were conducted across a broad frequency range (4–100 Hz). Statistical comparisons between conditions (AVS, rTMS + AVS, TBS, and sham rTMS) were performed using non-parametric repeated-measures one-way ANOVA with a two-tailed significance threshold of α < 0.01. When a significant cluster was identified, pairwise permutation tests were conducted between each active condition and the sham control to identify the source of the effect. Statistically significant clusters were defined as those comprising at least two neighbouring EEG channels.

## 3. Results

Data from 20 participants (80 sessions) were analysed (there were no dropouts). These included 12 males and 8 females, with a mean age of 23.1 ± 2.3 (range of 21 to 30). The mean RMT was 50.4 ± 6.5. Overall, all interventions were well-tolerated, with no serious adverse events reported. There was one transient self-resolving instance of mild nausea and dizziness during AVS, and an instance of mild fatigue following rTMS + AVS.

### 3.1. Event-Related Synchronisation/Desynchronisation

**Figure 2:**
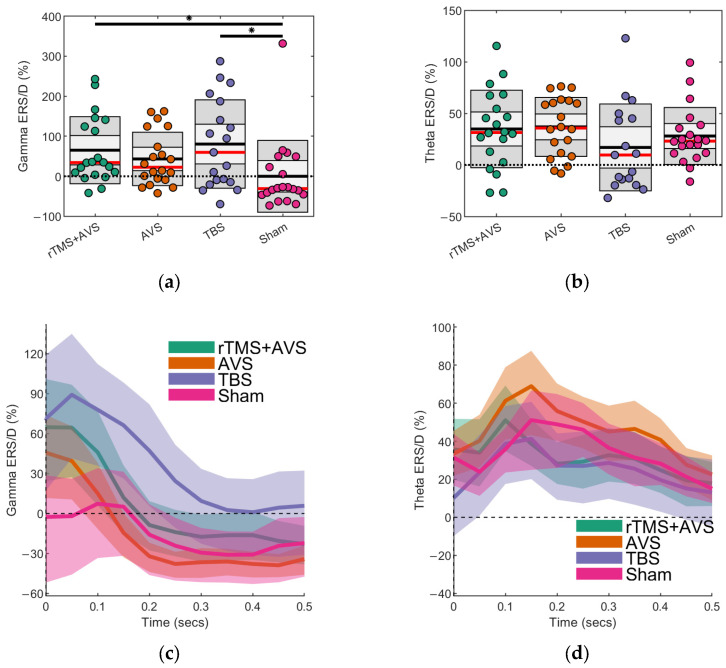
Evoked theta and gamma oscillatory activity following picture onset. Event-related synchronization/desynchronization (ERS/D) is presented as a proportion of oscillatory power relative to eyes-open resting state obtained prior to any intervention. Scatter plots show the mean (black line), median (red line), and the 95% confidence interval (dark grey region). Black bars with an asterisk indicate that the difference between two conditions is statistically significant (*p* < 0.05): (**a**) Gamma (38–42 Hz) activity at the region of interest (Oz); (**b**) Theta (4–8 Hz) activity at the region of interest (C1, C3, CP1, and CP3); (**c**) Average gamma ERS/D over time displayed with bootstrapped 95% confidence intervals; (**d**) Average theta ERS/D over time displayed with bootstrapped 95% confidence intervals.

One gamma ERS value from a TBS session was identified as an outlier. MRMM analysis revealed a significant effect of Condition on gamma activity (*F*_(3, 60)_ = 3.88, *p* = 0.01; see Figure 2a,c). Post hoc pairwise comparisons of active conditions with sham showed significantly higher occipital gamma ERS for rTMS + AVS (Cohen’s *d* = 0.71; 95% CI = 0.17 to 1.24; *p* = 0.01) and TBS (Cohen’s *d* = 0.87; 95% CI = 0.32 to 1.41; *p* = 0.002). Though not statistically significant, gamma ERS numerically increased for AVS relative to sham (Cohen’s *d* = 0.47; 95% CI = −0.06 to 1.01; *p* = 0.09).

Three theta ERS values, all from TBS sessions, were identified as outliers, likely reflecting residual EEG artefacts associated with TMS in these participants, and were excluded from analysis. MRMM analysis revealed no significant effect of Condition on theta activity (*F*_(3, 60)_ = 2.18, *p* = 0.10; see Figure 2b,d). Effect sizes for the active conditions compared to sham were in the small-moderate range: AVS (Cohen’s *d* = 0.26; 95% CI = −0.27 to 0.78), rTMS + AVS (Cohen’s *d* = 0.20; 95% CI = −0.33 to 0.73), and TBS (Cohen’s *d* = −0.39; 95% CI = −0.95 to 0.16).

**Figure 3:**
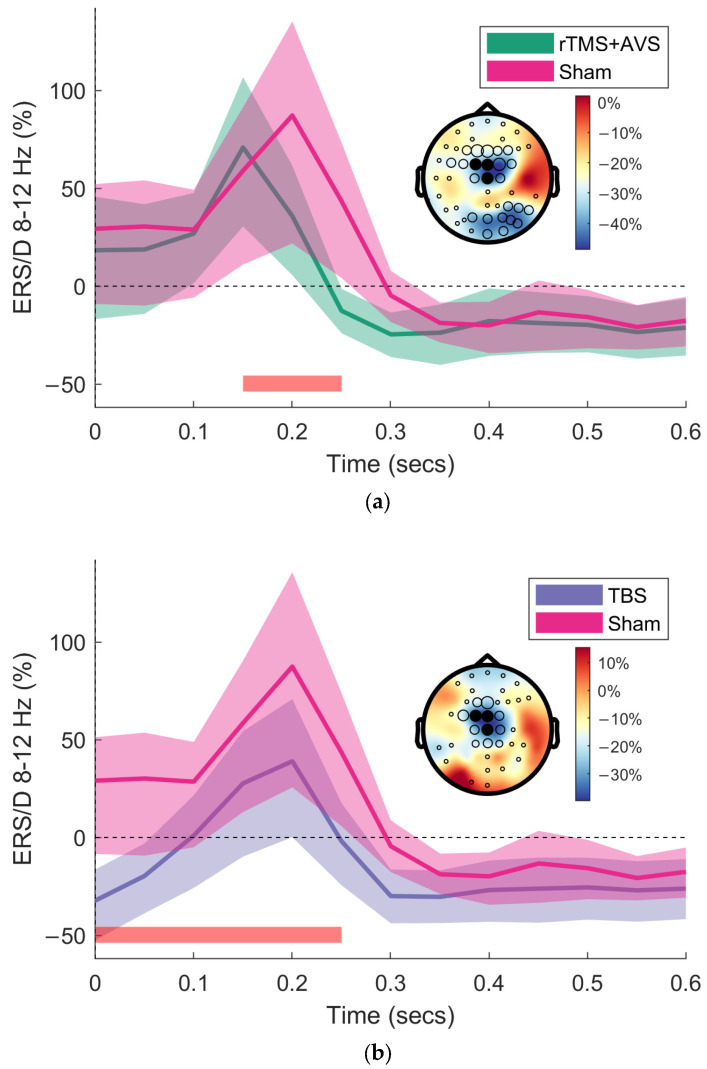
Cluster-based permutation testing of event-related synchronisation/desynchronisation (ERS/D). Time-frequency representations (TFRs) were generated by averaging brain activity following picture onset during the picture viewing portion of the recognition memory task. Brain activity is shown at midline channels (Cz, FCz, FC1) for the alpha frequency band (8–12 Hz). TFRs are displayed with bootstrapped 95% confidence intervals. Topographical plots are of the difference between active conditions (rTMS + AVS and TBS) and sham. Black circles indicate channels with a significant difference, with circle size proportional to the number of significant samples. Filled black circles denote the region of interest for TFR plotting. The time interval for the significant cluster is indicated by the red bar: (**a**) rTMS + AVS significantly reduced alpha and low beta frequency band activity (7–22 Hz) compared to sham; (**b**) TBS significantly reduced theta, alpha and low beta (5–30 Hz) compared to sham.

In addition to our region-of-interest analyses, we conducted exploratory, data-driven analyses using cluster-based permutation testing to assess differences across all EEG channels, 0 to 1000 ms post-picture onset, and frequencies between 4–100 Hz. An initial one-way repeated measures ANOVA identified significant clusters, prompting pairwise comparisons for active conditions against sham. This identified a significant negative cluster of reduced alpha (8–12 Hz) and lower beta (13–25 Hz) activity in frontocentral channels (predominantly Cz, FCz, and FC1) within a 300 ms time interval following picture onset for TBS and rTMS + AVS conditions (Figure 3).

### 3.2. Phase-Amplitude Coupling

**Figure 4:**
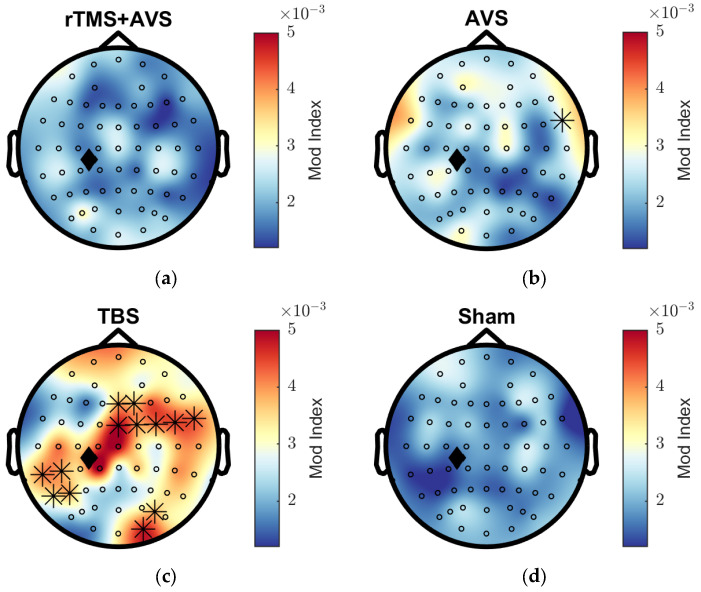
Theta-gamma phase-amplitude coupling across conditions. Theta-gamma phase-amplitude coupling was measured using the modulation index (MI). Topographical distributions show the MI for each condition during a post-picture onset time interval of 500 to 1850 ms. Black asterisks indicate channels that significantly differ for active conditions compared to sham, and the black diamond indicates the approximate TMS target location: (**a**) rTMS and AVS; (**b**) AVS; (**c**) TBS; (**d**) Sham rTMS.

To compare theta–gamma phase–amplitude coupling (PAC) between active and sham conditions, we used a data-driven permutation testing approach [62,63,64]. First, the modulation index (MI) was computed at all EEG electrodes for each condition. These values were then randomly permuted between each active condition and sham across 10,000 iterations to generate a null distribution from surrogate data. Electrodes where the observed MI in the active condition exceeded the 95% confidence interval of the null distribution are shown in Figure 4. The AVS condition increased MI at a right temporal electrode (FT8). TBS showed widespread increases in MI, including left temporoparietal regions (TP7, P7, CP5, P5), right parieto-occipital regions (O2, PO6), and right frontocentral regions (Fz, FCz, F2, FC2, FC4, FC6, FT8).

### 3.3. Recognition Memory Task Performance

There was no difference in performance between conditions for the recognition memory task (Table 1). Effect sizes for recognition accuracy (hits) for active conditions compared to sham were small: AVS (Cohen’s *d* = 0.02; 95% CI = −0.33 to 0.38), rTMS + AVS (Cohen’s *d* = 0.21; 95% CI = −0.15 to 0.57), and TBS (Cohen’s *d* = 0.09; 95% CI = −0.26 to 0.45).

## 4. Discussion

Neuronal entrainment within theta and gamma frequency bands has shown promise as a means to engage hippocampal network activity and enhance memory function. In this study, we investigated the effects of AVS, TBS, and a novel combination of synchronised rTMS with AVS, on frequency-specific brain activity and performance on a recognition memory task. Both TBS and the combined rTMS + AVS condition significantly increased occipital gamma-band activity during picture viewing. Additionally, cluster-based permutation testing revealed that these interventions reduced alpha and low-beta activity in frontocentral midline channels during the early (<300 ms) phase of picture presentation. TBS also enhanced theta–gamma phase-amplitude coupling across a broad region surrounding the stimulation site, including the left centroparietal, right frontocentral, and right parieto-occipital areas. Contrary to our hypothesis, however, we did not find any significant cognitive benefits in visual recognition memory performance for the active conditions compared to sham stimulation.

TBS increased gamma and reduced early alpha/beta activity. To the best of our knowledge, this is the first demonstration of neuronal entrainment during the TBS inter-train interval. Both increased gamma and reduced alpha/beta have previously been linked to enhanced memory performance. Specifically, gamma oscillations are associated with memory retrieval [17], suggesting TBS-induced gamma entrainment may support the consolidation and encoding of salient features in the presented stimuli. Alpha and beta desynchronisation support item recognition and lure discrimination, with stronger desynchronisation correlating with increased demands for memory specificity (i.e., stimulus details) [65]. Reductions in alpha and beta activity during stimulus presentation in associative memory tasks have been linked to memory formation processes [66], and are predictive of brain activity during episodic memory retrieval [67]. Together, these findings suggest that TBS may enhance neurophysiological processes underlying episodic memory.

However, it is important to note that we did not observe behavioural improvements in recognition task performance. The small effect sizes observed for recognition memory outcomes suggest that the absence of statistically significant differences is unlikely to be due to insufficient statistical power. Our study was powered based on Hermiller, Chen [26], which reported significant memory improvements using a similar TBS experimental paradigm. Methodological differences may underlie this discrepancy, including: (1) use of standardised MNI coordinates instead of individualised fMRI-guided TMS targeting; (2) use of a circular coil instead of a figure-of-8 coil, intended to compensate for spatial imprecision from group-based targeting by stimulating a broader cortical area; (3) use of a 40 Hz triplet frequency for TBS, selected to align more closely with the frequencies used during AVS; and (4) differences in experimental and control conditions between studies. Our findings suggest that measures of brain activity may be more sensitive markers of neural modulation than behavioural outcomes. Recognition memory tasks engage several cognitive processes, including attention, encoding, and recall. Behavioural outcomes therefore reflect the combined result of these processes. In contrast, ERS/D measures may reflect more discrete, individual processing stages, such as item recognition (alpha/beta ERD) and information encoding (gamma ERS). If TBS selectively modulates one of these stages, then the neurophysiological measures that capture them will be more sensitive to detect the effects compared to aggregate behavioural outcomes.

To the best of our knowledge, this is the first investigation of the novel combination of rTMS and AVS, demonstrating increases in occipital gamma as well as a reduction in frontocentral alpha activity. Contrary to our hypothesis, we did not find evidence to suggest that rTMS + AVS enhances theta-band activity, or recognition memory. Interestingly, both the pattern and magnitude of effects for this combined intervention closely resembled those obtained using TBS. Notably, participants spontaneously reported milder discomfort during rTMS sessions compared to TBS, suggesting that rTMS may offer a more tolerable and practical alternative for both research and clinical applications. Additionally, rTMS produced fewer EEG artefacts, further supporting its utility in studies requiring concurrent neurophysiological recording.

AVS did not significantly increase occipital gamma, although a numerical increase was observed, consistent with expectations based on prior literature, which have demonstrated this effect to be robust [68]. For example, Chan, Suk [45] reported single session increases in 40 Hz PSD ranging from 7.74 to 7.95 dB at occipital electrodes following AVS in both cognitively normal participants and individuals with mild Alzheimer’s disease. Importantly, these studies assessed entrainment during AVS (i.e., while the light and sound were presented), whereas our investigation focused on immediate post-stimulation effects. Indeed, comprehensive assessments of flickering light stimuli across various frequencies, colours, and luminance levels suggest that entrainment persists only weakly beyond the stimulation period, even under optimal conditions (i.e., white light, 400 cd/m^2^ intensity, flickering at 34–38 Hz) [69]. Although occipital gamma ERS was not observed, AVS did increase theta–gamma phase-amplitude coupling at a right frontotemporal site (FT8). However, this finding should be interpreted with caution. FT8 is a single electrode location that is prone to muscle artefacts and is anatomically distant from the visual cortex, where AVS effects are typically strongest. As such, while interesting, this result requires further validation in future studies with more robust spatial coverage and artefact control.

Potential limitations of the study include: (1) a relatively small sample of healthy participants. Although we retained sufficient statistical power by employing a crossover design, which reduced inter-individual variability, the exclusion of outliers due to rTMS-related EEG artefacts may have reduced statistical power for neurophysiological analyses; (2) Participants only received a single session, which likely contributed to the small effect sizes observed, consistent with meta-analytic findings in the rTMS literature [70,71]. Previous research has shown that three sessions of rTMS targeting the hippocampal network can significantly improve episodic memory [72]. These findings suggest that future investigations using repeated sessions of TBS as well as rTMS + AVS may be warranted considering significant neurophysiological effects observed in the present study.

## 5. Conclusions

In conclusion, the current study found that rTMS protocols, including TBS as well as the novel rTMS + AVS combination, increased gamma activity, and reduced alpha and lower beta activity relative to sham rTMS. Additionally, TBS further increased theta-gamma phase amplitude coupling. These neurophysiological changes have previously been associated with improved episodic memory performance in prior research, although notably no significant improvements were observed in recognition memory task performance in this study. Despite the absence of behavioural effects, the observed neural changes suggest that further research is warranted to explore the impact of repeated stimulation sessions on memory outcomes.

## Figures and Tables

**Table 1:** Recognition memory task performance. Values indicate means and SDs in parentheses.

	AVS	rTMS + AVS	TBS	Sham	*F*_(3, 60)_	*p*-Value
Recognition hits	0.70 (0.13)	0.72 (0.15)	0.71 (0.14)	0.69 (0.14)	0.55	0.65
Lure hits	0.73 (0.11)	0.70 (0.14)	0.68 (0.12)	0.72 (0.12)	2.07	0.11
Recognition D-prime	1.18 (0.51)	1.21 (0.56)	1.08 (0.44)	1.15 (0.37)	0.66	0.58
Recognition RT (ms)	1295 (242)	1362 (247)	1325 (216)	1295 (181)	0.94	0.43

## Data Availability

The raw data supporting the conclusions of this article will be made available by the authors on request.

## Abbreviations

The following abbreviations are used in this manuscript:

ANOVA	Analysis of variance
AVS	Audiovisual stimulation
EEG	Electroencephalography
ERS/D	Event-related synchronisation/desynchronisation
fMRI	Functional magnetic resonance imaging
HEAR	High-variance electrode artifact removal
HF-rTMS	High-frequency repetitive transcranial magnetic stimulation
ICA	Independent component analysis
MI	Modulation index
MRMM	Mixed-effect repeated measure model
PAC	Phase-amplitude coupling
RMT	Resting motor threshold
RS-EEG	Resting-state electroencephalography
rTMS	Repetitive transcranial magnetic stimulation
SOUND	Source-estimate-utilizing noise-discarding
STFT	Short-time Fourier transform
TBS	Theta burst stimulation
TFR	Time frequency representation
TMS	Transcranial magnetic stimulation
